# Polyimide-Based Flexible Microelectrode Array for Non-Invasive Transcorneal Electrical Stimulation

**DOI:** 10.3390/s25165198

**Published:** 2025-08-21

**Authors:** Víctor Manuel Carpio-Verdín, Natiely Hernández-Sebastián, Bernardino Barrientos-García, Silvia Solis-Ortiz, Erik R. Bojorges-Valdez, Francisco López-Huerta, Carlos Ismael Mares-Castro, Wilfrido Calleja-Arriaga

**Affiliations:** 1 Centro de Investigaciones en Óptica, A.C. Loma del Bosque 115, León 37150, Mexico; victorcv@cio.mx (V.M.C.-V.); bb@cio.mx (B.B.-G.); carlosmares@cio.mx (C.I.M.-C.); 2 Departamento de Ciencias Médicas, División de Ciencias de la Salud, Universidad de Guanajuato, 20 de enero 929, León 37320, Mexico; ms.solis@ugto.mx; 3 Departamento de Estudios en Ingeniería para la Innovación (DEII), Universidad Iberoamericana Ciudad de México, Ciudad de México 01219, Mexico; erik.bojorges@ibero.mx; 4 Facultad de Ingeniería Eléctrica y Electrónica, Universidad Veracruzana, Boca del rio, Veracruz 94294, Mexico; frlopez@uv.mx; 5 Instituto Nacional de Astrofísica, Óptica y Electrónica—INAOE, Puebla 72840, Mexico; wcalleja@inaoep.mx

**Keywords:** flexible electronic device, MEMS, microelectrode array, surface micromachining, transcorneal electrical stimulation

## Abstract

Transcorneal electrical stimulation (TES) is a promising treatment for several retinal degenerative diseases (RDDs). TES involves the application of a controlled electrical current to the anterior surface of the cornea, aimed at activating the retina and posterior ocular structures. Dawson–Trick–Litzkow (DTL) and ERG-JET electrodes are among the most widely used for TES. However, their continuous metallic surface design limits spatial resolution and the ability to perform selective ES. In this work, we present the development of a transcorneal electrical stimulation (TES) electrode that, unlike conventional electrodes, enables spatially selective TES. The proposed electrode design consists of an array of 20 independent microelectrodes distributed across the central and paracentral regions of the cornea. The fabrication process combines surface micromachining and flexible electronics technologies, employing only three structural materials: aluminum (Al), titanium (Ti), and polyimide (PI). This material selection is critical for achieving a simplified, reproducible, and low-cost fabrication process. The fabricated electrode was validated through electrical and electrochemical testing. The results show a relatively high electrical conductivity of Al/Ti structures, low electrochemical impedance values—ranging from 791 kΩ to 1.75 MΩ for the clinically relevant frequency range (11 to 30 Hz)—and a high charge storage capacity of 1437 mC/cm^2^. The electrode capacity for electrical signal transmission was demonstrated through in vitro testing. Finally, the applicability of the TES electrode for electroretinogram (ERG) recording was evaluated by measuring its optical transmittance across the visible wavelength range.

## 1. Introduction

The retina is a thin layer of neural tissue, approximately 0.5 mm thick, that lies on the back side of the ocular globe. Its main function is to convert light into electrical signals through the photoreceptor cells, enabling the generation and transmission of visual information to the brain [1,2].

Retinal degenerative diseases (RDDs) are a group of heterogeneous disorders characterized by progressive vision loss due to damage or degeneration of retinal cells, primarily the photoreceptors and the retinal pigment epithelium (RPE) [3,4]. The most prevalent RDDs include age-related macular degeneration (AMD), diabetic retinopathy (DR), and retinitis pigmentosa (RP). Collectively, these conditions are responsible for more than 300 million cases of blindness and visual impairment worldwide [3,5,6].

Currently, there is no effective treatment that fully slows or reverses the pathological process of these diseases. Therefore, the reported therapies primarily focus on treating symptoms and delaying disease progression [5,7]. For instance, nutritional supplements, such as vitamins and antioxidants, have been used to mitigate cellular damage associated with AMD and RP [5,7,8]; anti-vascular endothelial growth factor (anti-VEGF) intravitreal injections are a therapeutic option to inhibit retinal neovascularization and vascular leakage in patients with wet AMD and DR; and laser treatments are employed in some cases to seal abnormal blood vessels and reduce visual damage [5,7,8,9,10].

Although these treatments have proven to be beneficial, their efficacy varies significantly among patients due to factors such as disease state, individual response to treatment, and the presence of comorbidities [7,8,9,10]. This variability has driven research into novel treatments aimed at protecting, restoring, and replacing retinal cells. Among the most promising strategies are gene therapy, retinal prosthetics, and electrical stimulation (ES) [5,7,8,9,10,11,12].

In particular, TES has gained increasing interest due to its potential to preserve and enhance the visual function in patients with RDDs [12,13,14,15]. This minimally invasive approach involves applying a controlled electrical current to the anterior surface of the cornea to activate the retina and the posterior ocular structures [12,14,15,16]. Several studies have demonstrated that TES therapy protects retinal ganglion cells and photoreceptors, preventing degeneration processes [13,17,18,19]. Although the exact mechanism remains unclear, TES is thought to upregulate certain bioactive factors, such as insulin-like growth factor 1 (IGF-1), brain-derived neurotrophic factor (BDNF), and ciliary neurotrophic factor (CNTF), promoting neuronal preservation and homeostasis of the retinal microenvironment [15,17,18,19,20,21].

At present, the OkuStim^®^ system is the only commercially available alternative for performing TES [22,23]. This system is specifically designed for the treatment of RP and consists of three main components: a stimulation box, an adjustable frame that fits the patient’s face, and a standard Dawson–Trick–Litzkow (DTL) electrode that is placed on the lower eyelid to supply the electrical signal. Recent studies have demonstrated that the use of the OkuStim system is beneficial for treating RP [23,24,25]. In these studies, biphasic pulses of 5 ms duration at 20 Hz were used, with stimulation applied for 30 min weekly at 200% of the phosphene threshold (ranging from 0.1 to 3 mA) [23,24,25,26].

As in the case of the OkuStim^®^ system, most experimental TES studies report the use of a mono-element electrode, usually either a DTL or an ERG-JET electrode [27,28]. For instance, in [27,29], DTL and ERG electrodes were used to apply ES on the cornea of patients diagnosed with RP. In [27], a biphasic pulse train with a duration of 10 ms, frequency of 2 Hz, and current ranging from 0.5 to 1 mA was applied. On the other hand, in [29], a protocol using biphasic pulses of 2 ms duration, 20 Hz frequency, and current ranging between 200 and 400 µA was implemented. As can be observed, the signals used are similar; other studies employed both square and sinusoidal signals, with frequencies up to 20 Hz, and currents ranging from 10 µA to 1 mA [30,31,32,33]. Although these studies have demonstrated that TES therapy can produce beneficial effects on visual perception, the electrodes employed have certain limitations; for example, they have restricted spatial resolution and are unable to perform selective ES. These constraints are particularly relevant in the treatment of conditions involving either central, peripheral, or total vision loss, where the ability to selectively target specific retinal areas and independently modulate the stimulation signal could significantly enhance therapeutic outcomes by enabling a more personalized approach [12].

In this work, we present the design, fabrication, and characterization of a novel TES electrode for spatially selective electrical stimulation across the corneal layer. The proposed design integrates an array of 20 planar microelectrodes distributed within a 6.2 mm × 6.2 mm area, covering both central and paracentral corneal regions. The fabrication process, based on surface micromachining and flexible electronics techniques, develops a three-layer microdevice: a thick polyimide (PI) substrate, an Al/Ti double metallic layer, and a thin PI passivation film. This material selection not only enables a simplified and reproducible fabrication process but also allows a mechanically flexible and robust prototype with electrical and electrochemical properties suitable for TES.

## 2. Materials and Methods

### 2.1. Design

**Figure 1:**
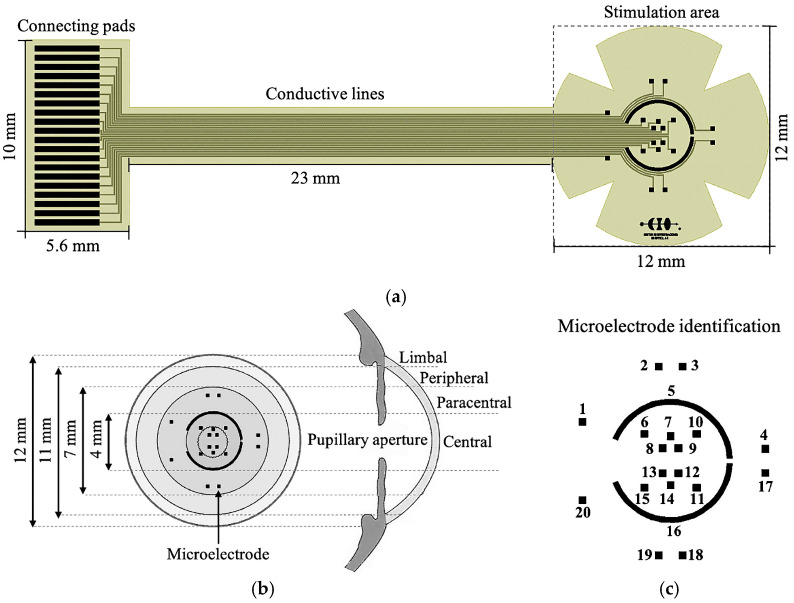
(**a**) Layout and geometric parameters of the proposed TES electrode, (**b**) distribution of the microelectrodes array in the central and paracentral region of the cornea, and (**c**) microelectrode identification.

The schematic of the proposed TES electrode is shown in Figure 1. As seen in Figure 1a, the TES electrode consists of three parts: the stimulation area, the conductive lines, and the connecting pads. The stimulation area shows an ergonomic design that allows attaching to the corneal curvature; its petal-shape structure integrates an array of 20 planar microelectrodes distributed around the central and paracentral regions of the cornea (see Figure 1b): 18 square microelectrodes sites with a 250 µm × 250 µm size and two others with an arc geometry approaching 5500 µm length and 170 µm width. These arcuate electrodes can stimulate the boundary between the central and peripheral regions—a zone particularly relevant in electrophysiological studies due to its high density of nerve fibers derived from the trigeminal nerve—which may enhance the propagation of electrical signals to other regions of the eye.

The conductive lines are the metal conductors that connect each stimulating site to its corresponding connecting pad. Depending on the positioning of the microelectrode and the pad, the conductive lines vary in length, ranging from 29 mm to 37 mm. To ensure an overall electrical reliability, the width of these lines is ruled to 60 µm. The design of the connection pads considered the technical features of an FFC/FPC connector model 5051102091 (Molex, Wellington, CT, USA), which contains 20 connections with a 0.5 mm pitch. The TES electrode has a total length of 41.6 mm and a maximum width of 12 mm in the stimulation area (see Figure 1a).

The design of the electrode considers the use of an Al/Ti metallic bi-layer as the structural material for the three components of the electrode: connecting pads, conductive lines, and microelectrode array. The strategic deposition of this metallic bi-layer—Ti onto Al—not only enhances the electrical conductivity of the structures but also preserves the biocompatibility of the prototype.

### 2.2. Fabrication Process

**Figure 2:**
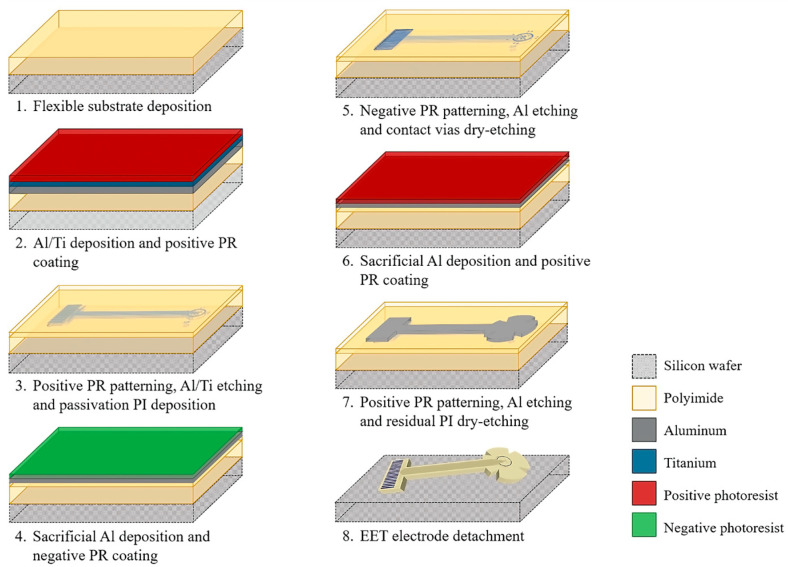
Schematic illustration of the fabrication process of the TES electrode.

Figure 2 shows the fabrication process of the proposed TES electrode. We used three masks and combined two manufacturing technologies: surface micromachine and flexible electronics. The process started by defining a 20 µm thick polymer layer as the flexible substrate (step 1, in Figure 2). Two combined polyimide films, PI-2610 and PI-2611 (HD MicroSystems™, Parlin, NJ, USA), were chosen as the substrate material due their relatively high mechanical flexibility and biocompatibility. In addition, the inherent properties of each type of PI were taken into account in their selection: PI-2610 provides strong adhesion to the silicon wafer (which functions as a mechanical support), whereas PI-2611 enables the deposition of thick films. To achieve a total thickness of 20 µm, the flexible substrate is formed by three deposition steps: first a PI-2610 layer, followed by two layers of PI-2611. Using a spin coater (Laurell Technologies Corporation, Lansdale, PA, USA), PI-2610 and PI-2611 were spun at 3000 revolutions per minute (rpm) and 2500 rpm, respectively, and consecutively cured at 350 °C for 2 h using a hot plate model HS40A (Torrey pines scientific, Carlsbad, CA, USA). After the last curing, the substrate surface was treated with oxygen plasma for 30 s using a PE-100 Reactive Ion Etch (RIE) system (Plasma Etch, Carson City, NV, USA). This treatment increases the film roughness, enhancing adhesion for subsequent metal film deposition.

Next, a structural Al/Ti bi-layer, with thicknesses of 150 nm and 50 nm, respectively, was deposited using an ATC Orion 8 sputtering system (AJA International, Inc., Hingham, MA, USA) (step 2, in Figure 2). Subsequently, a standard photolithography process was performed on the metallic bi-layer using a first-level mask: a 1.5 µm thick layer of positive AZ-1512 photoresist (PR) (Microchemical GmnH, Ulm, Germany) was spin-coated and baked at 90 °C, for 60 s. UV exposure was performed using a MA/BA GEN4 PRO series mask aligner (Süss MicroTec, Garching, Germany) for 4 s, and the photoresist patterns were developed using the AZ 726 MIF developer (MicroChemicals GmnH, Ulm, Germany) for 35 s. The metallic bi-layer was selectively etched in deionized water (DI)/hydrofluoric acid (HF)/nitric acid (HNO_3_) aqueous solution with a volume ratio of 20:1:1 for 23 s. The residual AZ-1512 PR layer was dissolved in acetone for 10 min using an ultrasonic cleaner (Elma Schmidbauer GmbH, Singen, Germany).

Afterwards, a passivation layer of PI-2610 was spun at 4000 rpm and cured at 350 °C for 2 h (step 3, Figure 2). The passivation layer was treated by oxygen plasma for 30 s using the RIE system, and a 100 nm thick sacrificial Al layer was deposited by the sputtering system. A standard photolithography process was performed on the Al film using a second level mask: a 1.2 µm thick layer of negative AZ-2020 PR (Microchemical GmnH, Ulm, Germany) was spin-coated and baked at 105 °C for 50 s (step 4, Figure 2). The negative PR was exposed to UV light for 10 s and developed in AZ 326 developer for 50 s. The sacrificial Al layer was then selectively etched using the commercial solution “Aluminum Etchant Type A” (Merk KGaA, Darmstadt, Germany) for 2 min. Subsequently, the passivation layer was selectively etched for 20 min with oxygen plasma to open the contact vias for the stimulation and pad sites (step 5, Figure 2). The residual sacrificial Al layer was etched using the Aluminum Etchant solution for 80 s at 40 °C.

To define the overall shape of the TES electrode, a 100 nm thick sacrificial Al layer was deposited, followed by a standard photolithography process using the positive PR AZ1512 and the third level mask (step 6, Figure 2). The sacrificial Al layer was selectively etched using the Al etchant solution for 80 s at 40 °C (step 7, Figure 2). Then, the residual PI was etched using oxygen plasm for 6 h. Finally, the flexible TES electrode was detached from the silicon wafer using DI water (step 8, Figure 2).

### 2.3. Electrode Characterization

#### 2.3.1. Electrical Testing

The I-V curve of each pad–microelectrode structure was measured using a Summit 12000 Probe Station (Cascade Microtech Inc., Beaverton, OR, USA) and a Keithley 4200A Parameter Analyzer (Tektronic Inc., Beaverton, OR, USA). The measurements were performed at room temperature using a two-probe configuration, where one of the probes was positioned on the connecting pad and the other on the corresponding microelectrode. A parameter analyzer was used to supply a sweep voltage from −5 V to 5 V, with steps of 0.01 V.

Considering these conditions, two types of measurements were conducted: one with the TES electrode placed on a flat substrate, and the other on a contact lens. In this way, it was not only possible to evaluate the electrical conductivity and resistivity of each pad–microelectrode structure, but also to assess the integrity of the structures under conditions similar to the operating ones.

#### 2.3.2. Electrochemical Characterization

**Figure 3:**
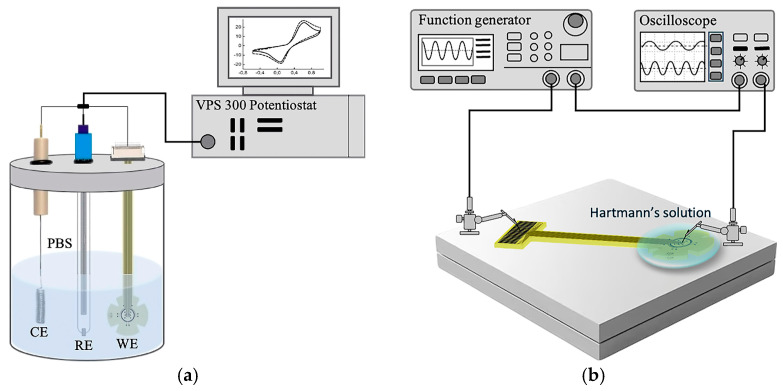
(**a**) Diagram of the three-electrode electrochemical system and (**b**) in vitro setup used for evaluating the electrical transmission performance of the microelectrode array.

The electrochemical performance of the planar microelectrodes was evaluated using an electrochemical impedance spectroscopy (EIS) and a cyclic voltammetry (CV) technique. These techniques were realized by means of a three-electrode system and a VSP-300 potentiostat/galvanostat (BioLogic, Seyssinet-Pariset, France), as shown in Figure 3a. In this arrangement, a platinum (Pt) wire was used as the counter electrode (CE) to complete the electrical circuit by allowing current to flow; a cylindrical silver/silver chloride (Ag/AgCl) electrode functioned as the reference electrode (RE), providing a stable and known potential; and the fabricated electrode array was utilized as the working electrode (WE), where the electrochemical reactions of interest occur and impedance is measured. To carry out the measurements of EIS and CV, the WE was connected to an FFC/FPC connector model 5051102091 (Molex, Wellington, CT, USA), and the three electrodes were immersed in a phosphate-buffered saline solution (PBS, 0.01 M, pH = 7.4) at room temperature.

The impedance spectra were recorded under open-circuit conditions using a 1–100 Hz frequency range with a 10 mV rms sine wave. Measurements were performed for each of the 20 microelectrodes, and the average impedance value was calculated and plotted.

The CV measurements were performed by applying a voltage sweep from −0.6 V to 0.6 V at a scan rate of 100 mV/s. The resulting curve was used to calculate the charge storage capacity of the microelectrode by integrating the area under the voltammograms.

#### 2.3.3. Electrical Signal Transmission

To evaluate the performance of the microelectrode array for transmitting electrical signals, the experimental setup shown in Figure 3b was used. The TES electrode was placed on a flat surface, and a few drops of Hartmann’s solution were poured on the stimulation site; this solution is used to simulate physiological conditions. A Hewlett 33120A function generator (Keysight Technologies, Inc., Santa Rosa, CA, USA) was used to supply a sinusoidal signal with a frequency of 11 Hz, a period of 10 ms, and a peak-to-peak voltage of 1 mV to one of the connecting pads.

The electrical signal was recorded using a Tektronix TDS 2004 oscilloscope (Tektronic Inc., Beaverton, OR, USA) under two experimental conditions: (i) direct electrical contact with the microelectrode and (ii) indirect contact, i.e., contact through the Hartmann’s solution (1 mm and 2 mm away from the microelectrode). This approach not only permits the evaluation of each pad–microelectrode structure’s ability to conduct an electrical signal, but also facilitates the analysis of its performance for transmitting the electrical signals through the biological media. This is particularly relevant for TES applications, where a tear film is the transmission medium between the electrode and the corneal surface.

#### 2.3.4. Transmittance Spectra Measurement

The optical transmittance of the flexible microelectrode array was measured using a SpectraRay/4 spectroscopic ellipsometer across a wavelength range from 250 nm to 1050 nm. This assessment reveals the capacity of the flexible polyimide substrate to transmit white light and also to determine the level of any blockage of the light by the microelectrode structures. Transmittance spectra were obtained separately for the flexible electrode (around the stimulation area) and the polymethyl methacrylate (PMMA) contact lens. This analysis provided insight into the optical transparency of the prototype, an essential requirement for applications such as ERG recordings.

## 3. Results and Discussion

### 3.1. Planar Electrode Array Fabrication

**Figure 4:**
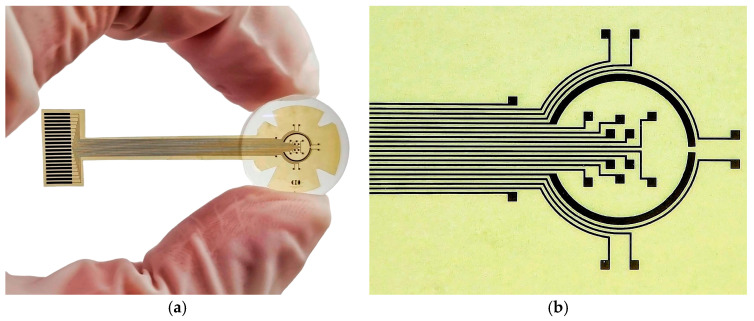
(**a**) Fabricated flexible TES electrode and (**b**) magnified image of the stimulation area.

Figure 4a shows the fabricated TES electrode, and Figure 4b shows a magnified view of the stimulation area. The resulting prototype exhibits spatial selectivity, mechanical flexibility and structural integrity. Furthermore, the use of only three structural layers (thick PI substrate, double metal, and PI passivation layer) allowed us to develop a simplified, reproducible, and low-cost fabrication process.

**Figure 5:**
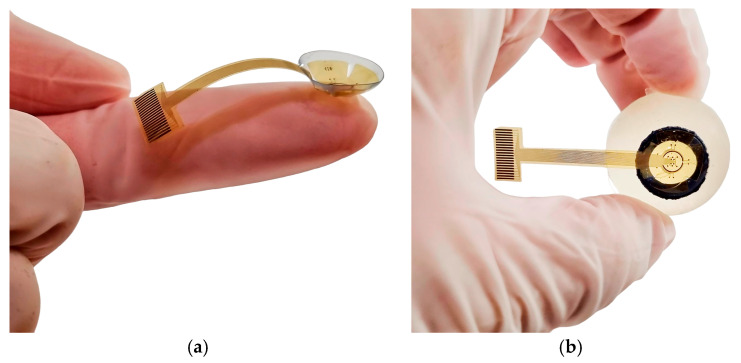
(**a**) The TES electrode is attached to a soft contact lens; and (**b**) the assembled system is placed onto the corneal surface of a human eye model.

The stimulation area of the fabricated TES electrode was attached into a PMMA contact lens (see Figure 5a). The incorporation of a petal-like structure in the electrode design, combined with the mechanical flexibility of the prototype, enabled optimal fitting into the concave lens. Then, to mimic physiological operating conditions, the assembled system was mounted onto the front surface of a human eye model, as shown in Figure 5b. The system seamlessly conforms to the corneal surface, ensuring stable placement and effective integration.

### 3.2. Electrical Testing

The TES electrode was designed to exhibit symmetry (see Figure 1a,c); therefore, we show results for only ten distinct pad–microelectrode configurations, identified as 1 to 10 in Figure 1c. These configurations differ in the length of the conductive lines (ranging from 31.2 mm to 36.5 mm) and in the microelectrode geometry (square and arc). For the squared microelectrodes, analytical calculations indicated that the shortest structure exhibited an electrical resistance of 98.74 Ω, increasing to 112.85 Ω for the longest structure. In the case of the pad–arc microelectrode, it exhibited an electrical resistance of 99.2 Ω.

Experimental measurements showed electrical resistance values of 98.27 Ω and 114.48 Ω for the shortest and longest square microelectrode structures, respectively, and 99.32 Ω for the arc microelectrodes. These results are consistent with the analytical predictions, exhibiting a maximum deviation of ±2 Ω. This small difference may arise from minor variations of the fabrication process. It is worth noting that the electrical resistance of the pad–arc microelectrode structure is similar to that obtained for the shortest pad–microelectrode structure, as the overall resistance is mainly determined by the conductive line rather than the microelectrode size.

**Figure 6:**
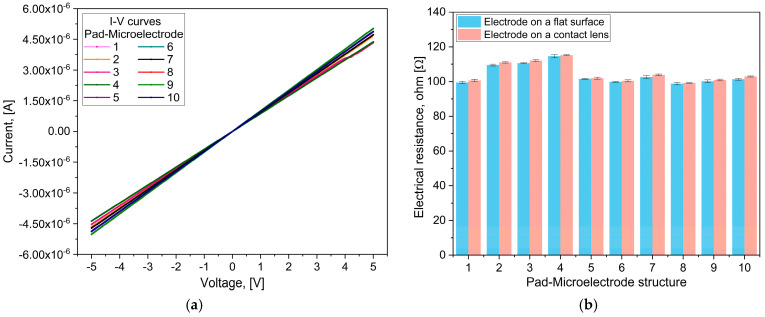
(**a**) I–V curves of each pad–microelectrode structure and (**b**) comparison of the pad–microelectrode’s electrical resistance when placed on a flat surface and when mounted on the contact lens.

Figure 6a presents the current–voltage (I–V) characteristics of the ten pad–microelectrode structures. We notice a linear behavior of the resistive elements and similar slopes of the curves due to their comparable lengths.

Figure 6b shows a comparison of the measured resistance for each pad–microelectrode when the TES electrode is placed on a flat surface and when it is mounted on the contact lens. A difference of about 1.5 Ω is observed. This reduced change does not significantly affect the prototype’s functionality nor its capacity to maintain stable electrical performance under physiological conditions. Additionally, error bars for each structure (n = 5 measurements) are included; the average uncertainty is 0.57 Ω; this relatively minor difference reflects the high reproducibility of the fabrication process.

### 3.3. Electrochemical Characterization

**Figure 7:**
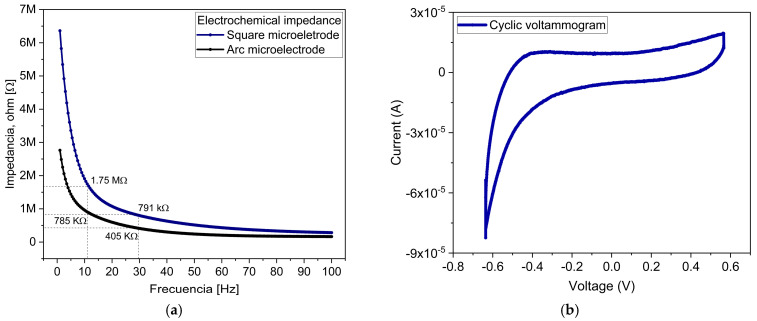
(**a**) Average electrochemical impedance spectroscopy and (**b**) cycle voltammogram measurements of Al/Ti microelectrodes.

Figure 7a shows the EIS data obtained for the microelectrode array. The average impedance reveals a decaying exponential frequency behavior, decreasing from 6.36 MΩ at 1 Hz to 284 kΩ at 100 Hz for the square microelectrodes, and from 2.76 MΩ at 1 Hz to 165 MΩ for the arc microelectrodes. This behavior reflects a dominant capacitive contribution by the microelectrode–electrolyte interface. Within the clinically relevant frequency range for TES (11 Hz to 30 Hz), the impedance of the square microelectrodes varies from 1.75 MΩ to 791 kΩ, while that of the arc microelectrodes ranges from 785 kΩ to 405 kΩ. These impedance values are consistent with the values reported for ES microelectrodes, which typically operate at low currents (100 µA to 500 µA) and at a few hertz within the frequency range [34,35,36,37].

The average CV behavior obtained for the microelectrode array is presented in Figure 7b. The area enclosed by the curve corresponds to the charge storage capacity (CSC). The storage capacity was determined by integrating the cathodic current over the voltage range and dividing the results by the scan rate; the average value for this parameter, obtained for the Al/Ti microelectrodes, was 1437 mC/cm^2^, which is superior to that of many ES microelectrode materials, including Ti/Pt [38,39], gold (Au) [40], titanium nitride (TiN) [36], and chrome (Cr)/Au [41]. In addition, both the CSC value and voltage range (ΔV=1.2 V) can be used to evaluate the ES signal voltage. For TES, typical values of the current are within the range from 100 µA to 500 µA; in the present case, the obtained value of the TES voltage is around 0.5 V [42]. This shows that the voltage value is within the compliance limits of TES.

### 3.4. Electrical Signals Transmission

**Figure 8:**
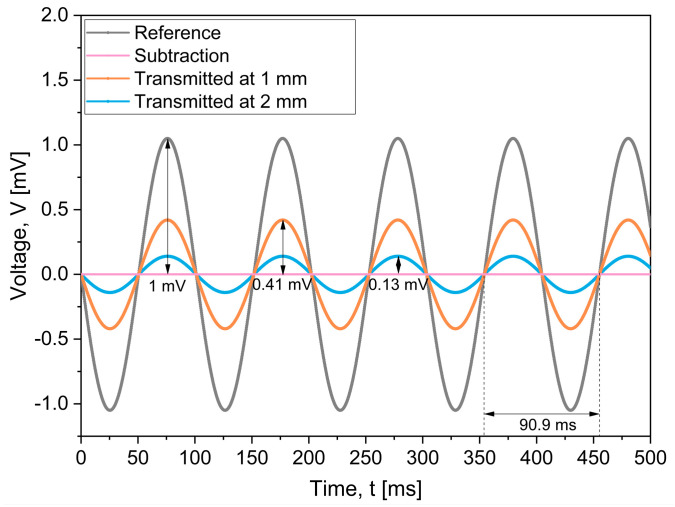
Comparison of the input signal (gray curve) with signals transmitted by the stimulating array. The pink curve represents the input signal minus the average transmitted signal from 20 microelectrodes (contact condition). The orange and blue curves correspond to the transmitted signal at 1 mm and 2 mm from the electrode surface, respectively.

Figure 8 shows the average result for the performance of the signals transmitted by each microelectrode under both direct and indirect contact conditions. The grey curve corresponds to the input signal (11 Hz and 1 mV peak-to-peak value), while the orange and blue curves correspond to the transmitted signals at distances of 1 mm and 2 mm from the stimulating sites, respectively. Under direct contact conditions, the transmitted signal from all 20 microelectrodes closely matched the input waveform in amplitude (1 mV), period (90.9 ms), and phase. To verify the fidelity of signal acquisition, the average transmitted signals was subtracted from the input signal, resulting in a flat residual curve (pink curve), which indicates a high degree of similarity. These results confirm precise signal transmission through the fabricated stimulating sites.

Under non-contact conditions, the electrical signals yield amplitudes of 0.41 mV and 0.13 mV, respectively. Despite the observed attenuation, the transmitted waveforms preserved both the period and phase of the input signal. This was further corroborated by Lissajous curves, which showed a 1:1 frequency ratio and a 0° phase shift between the input and transmitted signals; these features reflect the fact that the transmitted signal was not distorted by any structural and functional defects in the TES electrode.

The observed reduction in signal amplitude is attributed to the electrochemical impedance at the microelectrode–saline solution interface. These results therefore confirm the capability of the microelectrode array to transmit electrical signals with high fidelity under non-contact conditions.

To ensure reliability and agreement in the measurements of electrical indirect conduction of the microelectrode array, we calculated the interclass correlation coefficient (3,3), given a value of 0.97 (with the parameter of probability *p* being practically 0 and the confidence level at 99.7%), which suggests strong consistency and agreement among measurements.

### 3.5. Transmittance Spectra Measure

**Figure 9:**
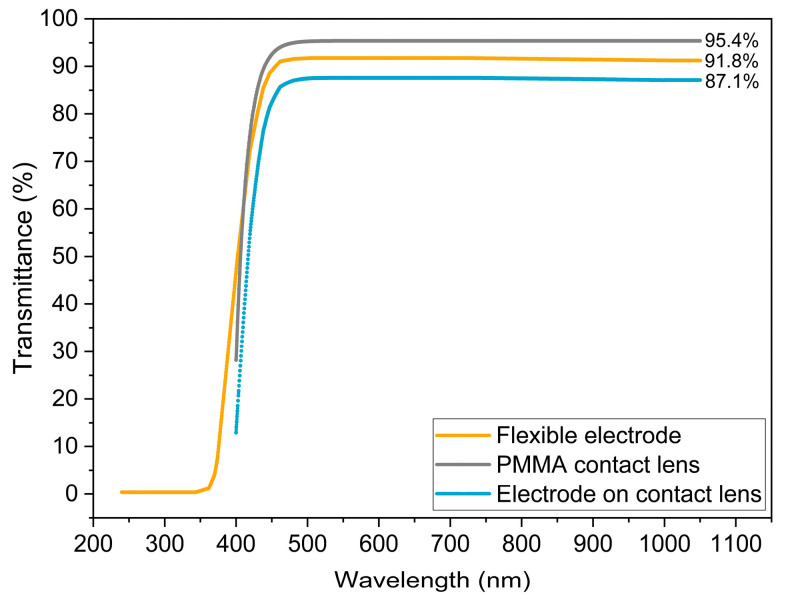
Transmittance spectra for flexible electrode, PMMA contact lens, and the electrode mounted on a contact lens.

Figure 9 presents the transmittance spectra obtained for the flexible electrode and the PMMA contact lens. The flexible electrode presents a constant value of transmittance of 91.8% for wavelengths greater than 400 nm. This implies that the microelectrode array structures do not significantly absorb light through the polyimide substrate. The PMMA contact lens shows a transmittance value of 95.4% in the visible spectrum range.

The transmittance of the combination of the electrode and the contact lens is calculated as the product of the two latter curves and shows a plateau value of 86.1%. Although further studies are needed to fully explore the potential of the fabricated microelectrode array for electrical signal recording, the transmittance values obtained indicate that it is suitable for ERG recording, where a white light pulse is supplied to the retina through the cornea.

### 3.6. Comparison Analysis

Table 1 presents a review of reported works dealing with TES by considering various types of electrode technologies. The comparison includes key features, such as electrode type, application, operating frequency, fabrication technology and stimulation corneal regions.

As observed from Table 1 and mentioned previously, the DTL and ERG-JET are the two types of electrodes most frequently reported in the literature. In addition, most recent works on selective TES include only numerical simulations. The proposed work aims to contribute to this subject, but departing from a different perspective: using a microelectrode array in order to selectively stimulate particular regions of the cornea. In addition, the use of advanced technologies—such as MEMS and flexible electronics—over conventional approaches enable the development of mechanically flexible, miniaturized, robust and highly efficient electrodes that provide structural variety and seamless integration with soft biological tissues.

## 4. Conclusions

A novel flexible electrode for spatially selective TES was designed, fabricated, and evaluated. The TES electrode consists of an array of 20 independent microelectrodes, ergonomically designed to conform to the corneal curvature and disposed to stimulate the central and paracentral regions of the cornea surface. It can be easily adapted to target either the peripheral or entire corneal area, depending on specific stimulation requirements.

A simplified, reproducible and low-cost fabrication process was achieved by combining surface micromachine and flexible electronics technologies and by employing only three structural materials: Al, Ti, and PI. The resulting device exhibited mechanical flexibility, structural integrity, and robustness.

Electrical and electrochemical characterizations reveal high electrical conductivity and efficient charge transfer of the electrode with values comparable to, or exceeding, those of state-of-the-art electrodes used in ES.

Additionally, in vitro testing validated the capability of the fabricated electrode to reliably transmit electrical signals through a physiological interface. This result, combined with the ability to transmit light in the visible spectrum, supports not only its potential for TES but also for recording electrical signals, as in ERG measurements.

## Figures and Tables

**Table 1:** Comparison analysis of reported works.

Reference	Electrode Type	Application	TES Parameters	Fabrication Technology	Stimulation Corneal Regions
This work	Microelectrode array	Selective TES	11 Hz, 10 ms, and 500 µA	MEMS and flexible electronics	Central and paracentral
[22,23]	DTL	TES	Bipolar rectangular pulses, 5 ms, 20 Hz, and 600 µA	Conventional	Inferior
[34]	DTL	TES	Biphasic rectangular pulses, 1 ms, 20 Hz, and 200 to 400 µA	Conventional	Inferior
[35]	DTL	TES	1 ms, 20 Hz, and 0.1–1 mA	Conventional	Inferior
[36]	ERG-JET	TES	6 Hz, 20–150 µA, and 10 ms	Conventional	Peripheral
[37]	ERG-JET	TES	Biphasic stimulus pulses, 6 Hz, 100–200 µA, and 10 ms	Conventional	Peripheral
[43]	Microelectrode array	Selective TES	Sinusoidal signal, 1–8 kHz and 1–100 mA	Numerical simulation	Several regions

## Data Availability

The data that support the findings of this study are available from the corresponding author upon request.

## Abbreviations

The following abbreviations are used in this manuscript:

TES	Transcorneal electrical stimulation
Al	Aluminum
Ti	Titanium
PI	Polyimide
rpm	revolutions per minute
ERG	Electroretinogram
RDDs	Retinal degenerative diseases
RPE	Retinal pigment epithelium
AMD	Age-related macular degeneration
DR	Diabetic retinopathy
RP	Retinitis pigmentosa
Anti-VEGF	Anti-vascular endothelial growth factor
ES	Electrical stimulation
IGF-1	Insulin-like growth factor 1
BDNF	Brain-derived neurotrophic factor
CNTF	Ciliary neurotrophic factor
DTL	Dawson–Trick–Litzkow
RIE	Reactive Ion Etch
PR	Photoresist
DI	Deionized water
HF	Hydrofluoric acid
HNO_3_	Nitric acid
EIS	Electrochemical impedance spectroscopy
CV	Cyclic voltammetry
CE	Counter electrode
Pt	Platinum
Ag	Silver
AgCl	Silver chloride
RE	Reference electrode
WE	Working electrode
PBS	Phosphate-buffered saline solution
PMMA	Polymethyl methacrylate
Au	Gold
TiN	Titanium nitride
CSC	Charge storage capacity
Cr	Chrome
